# REGIONAL VARIATIONS IN INFANT AND CHILD MORTALITY IN NIGERIA: A MULTILEVEL ANALYSIS

**DOI:** 10.1017/S0021932013000734

**Published:** 2014-01-10

**Authors:** SUNDAY A. ADEDINI, CLIFFORD ODIMEGWU, EUNICE N. S. IMASIKU, DOROTHY N. ONONOKPONO, LATIFAT IBISOMI

**Affiliations:** * Demography and Population Studies Programme, Schools of Public Health and Social Sciences, University of the Witwatersrand, Johannesburg, South Africa; † Demography and Social Statistics Department, Obafemi Awolowo University, Ile-Ife, Nigeria; ‡ Department of Geography, University of Zambia, Lusaka, Zambia; § Department of Sociology and Anthropology, University of Uyo, Nigeria

## Abstract

There are substantial regional disparities in under-five mortality in Nigeria, and evidence suggests that both individual- and community-level characteristics have an influence on health outcomes. Using 2008 Nigeria Demographic and Health Survey data, this study (1) examines the effects of individual- and community-level characteristics on infant/child mortality in Nigeria and (2) determines the extent to which characteristics at these levels influence regional variations in infant/child mortality in the country. Multilevel Cox proportional hazard analysis was performed on a nationally representative sample of 28,647 children nested within 18,028 mothers of reproductive age, who were also nested within 886 communities. The results indicate that community-level variables (such as region, place of residence, community infrastructure, community hospital delivery and community poverty level) and individual-level factors (including child's sex, birth order, birth interval, maternal education, maternal age and wealth index) are important determinants of infant/child mortality in Nigeria. For instance, the results show a lower risk of death in infancy for children of mothers residing in communities with a high proportion of hospital delivery (HR: 0.70, *p*<0.05) and for children whose mothers had secondary or higher education (HR: 0.84, *p*<0.05). Although community factors appear to influence the association between individual-level factors and death during infancy and childhood, the findings consistently indicate that community-level characteristics are more important in explaining regional variations in child mortality, while individual-level factors are more important for regional variations in infant mortality. The results of this study underscore the need to look beyond the influence of individual-level factors in addressing regional variations in infant and child mortality in Nigeria.

## Introduction

Despite modest improvements in child health outcomes during the 20th century, infant and child mortality rates remain unacceptably high in the sub-Saharan African countries. Globally, around 7 million under-five deaths were recorded in 2011 (UNICEF, 2012). Sub-Saharan Africa is a major contributor to this statistic as more than two in five under-five deaths occur in the region (Black *et al.*, 2003; Rutherford *et al.*, 2010). Scholars have attempted to examine the factors influencing this. For instance, studies have established a significant relationship between infant and child mortality and individual-level characteristics such as maternal education, wealth status and other socioeconomic characteristics (Adetunji, 1995; Zaba & David, 1996; Lawoyin, 2001; Buor, 2002; Odimegwu, 2002; Fayeun & Omololu, 2011). Other studies have shown that attributes of the community context tend to influence the health outcomes of individuals (Sastry, 1996; Uthman, 2008; Babalola & Fatusi, 2009; Adekanmbi *et al.*, 2013). This suggests that living in an economically and socially deprived community or neighbourhood could bring about an increase in mortality risk.

Nigeria – the most populous country in Africa – is characterized by socially and economically advantaged and disadvantaged regions. The country is geographically, religiously, socially, ecologically and economically diverse. This has led to varied disease exposures and different health outcomes (Wall, 1998; Lawoyin, 2001; Grais *et al.*, 2007). From the arid northern region of the country to the savannah west, from the predominantly Islamic north-west to the south-east vastly dominated by Christians, the country is highly heterogeneous and diverse. As a result, there is a huge diversity in the regional environment, cultural practices (Antai *et al.*, 2009), health-seeking practices (Babalola & Fatusi, 2009), socioeconomic status (Aremu *et al.*, 2011) and the political milieu.

To date, Nigeria has a very high rate of under-five mortality, as about one in every six children born in the country dies before the age of five (NPC & ICF Macro, 2009). Worse still, infant and child mortality rates vary substantially from one region of the country to the other. For instance, under-five mortality rate ranges from 89 per 1000 live births in the south-west to 222 per 1000 live births in the north-east. Besides, Nigeria is not making sufficient progress towards the attainment of Millennium Development Goal four (MDG-4), as the under-five mortality rate in the country for the 1998–2003 period was 201 per 1000 live births, while the rate marginally declined to 157 per 1000 live births during the 2004–2008 period (NPC & ORC Macro, 2004; NPC & ICF Macro, 2009). In addition, as noted earlier, Nigeria is by far the most populous country in Africa and has a very huge childhood population. According to the 2006 population and housing census, Nigerian's population is 140,431,790 and the population of the under-five children is 16.1% of the total population (NPC & ICF Macro, 2009).

Furthermore, despite the improvement in medical technology, reports by NPC & ICF Macro (2009) indicate that Nigeria is still faced with several health challenges. These include high rates of under-five mortality (157 per 1000 live births), high teenage pregnancy (23% of young women aged 15–19 have already given birth), many poor pregnancy outcomes (such as stillbirth, spontaneous abortion and low birth weight), poor survival chances for the newborn and high unmet need for family planning (20% of currently married women have an unmet need for contraception in the country). Also, the percentage of births assisted by unskilled birth attendants is 60% in the country. The list of the country's public health problems is endless.

While many studies have been conducted on childhood mortality in Nigeria, as in other developing countries, Nigerian studies have paid more attention to the influence of individual-level attributes, and less attention to community-level determinants of childhood mortality. However, the literature shows that knowledge about the determinants of childhood mortality at the individual level is insufficient to address the problem (Sastry, 1996; Whitworth & Stephenson, 2002; Griffiths *et al.*, 2004; Harttgen & Misselhorn, 2006; Antai, 2011b). This is because the contextual characteristics of the community or neighbourhood where children are born or raised tend to modify child-level and mother-level characteristics and therefore affect children survival chances. In addition, past Nigerian studies that have examined community determinants of child survival have focused mainly on ages 0–59 months, thereby ignoring what the effects of community characteristics on child survival are during infancy (i.e. age 0–11 months) and childhood (12–59 months). To this end, this study aims to (1) examine the effects of individual- and community-level characteristics on child survival during infancy and childhood, and (2) determine the extent to which characteristics at the individual and community levels explain regional variations in infant and child mortality in Nigeria.

## Theoretical background

From a theoretical standpoint, Mosley and Chen's 1984 model on the proximate causes of childhood mortality establishes a relationship between child survival and determinants at various levels of operation: individual, household and community levels (WHO, 2003). Diez-Roux *et al.* (2001) posited that the physical and social characteristics of the neighbourhood where a person lives may affect health and health-related behaviour. Galster's (2010) work on the mechanisms of neighbourhood effects theory observed a link between residential environment and the health outcomes of individual adults and children residing in such environment or community.

The effect of community context, where children are born or raised, on their survival chances has been widely recognized (Sastry, 1997b; Omariba *et al.*, 2007; Antai, 2009). Evidence suggests that living in an economically and socially deprived community or neighbourhood is associated with an increased risk of under-five mortality (Aremu *et al.*, 2011). For instance, children born or raised in a community that lacks electricity, good drinking water and health facilities are likely to suffer from the same deprivation that can directly or indirectly influence their health outcomes. Further, Manda (2001) opined that Demographic and Health Surveys (DHSs) often collect birth history data that are clustered at the household and community levels. Sastry (1997b) also maintained that most demographic surveys conducted in developing countries often collect survival data that are clustered at both family and community levels. Omariba and colleagues (2007) argued that since DHSs collect child survival data from individual mothers in sampled households, then the children of those mothers cannot be regarded as independent observations. This is as a result of natural clustering or as a result of the data collection procedures (i.e. two-stage cluster sampling design) used in DHS data collection (Sastry, 1997b). The children from sampled households who are also nested within the same individual mother's level tend to share similar characteristics and common genetic factors. This is also true of children from the same community. Individuals in the same community are likely to be more homogenous than those from different communities. Similarities are expected in the health outcomes of children who are exposed to the same environmental conditions. By contrast, differences are expected in health outcomes of children raised in different communities due to differences in community characteristics (Harttgen & Misselhorn, 2006).

A distinction is drawn between children living in a relatively better-off neighbourhood and those living in a relatively worse-off neighbourhood (Macintyre *et al.*, 2002). Children living in two different households with similar socioeconomic characteristics can suffer different mortality risks if they are from two contrasting communities. Sastry (1996) contended that community characteristics can exacerbate or mitigate mortality risks of individuals depending on the environmental context the individuals find themselves. Griffiths (2004) also argued that community services and levels of infrastructural development of a community are capable of amplifying or reducing mortality risks among the children. This is because an individual child resident in a household unit, which in turn is located within a community, is exposed to various levels (within the societal hierarchy) that either directly or indirectly influence his or her survival chances.

In addition, Whitworth & Stephenson (2002) maintained that two neonates with similar characteristics may suffer different neonatal mortality risks because of the community contextual effects. The authors argued that these differentials in mortality risks may be as a result of differences in the provision of antenatal and obstetric health care or the effects of environmental conditions the children are exposed to. Also, individuals residing in the same community tend to share similar preferences, cultural practices, values and customs. The reason is that individuals with similar tastes and values tend to cluster and live together (Sastry, 1997a). All this clustering and living together of people with common norms, values, identities and cultural practices, as well as spatial inequality in infrastructural development (Antai, 2011b), has a direct or indirect effect on the health outcomes of under-five children and this often brings about differentials in health outcomes between communities, particularly communities with contrasting characteristics.

Motivated by the emerging interest in the study of effects of community or neighbourhood context on health outcomes in developing countries, this paper seeks to examine the extent to which characteristics at individual and community levels influence regional variations in infant and child mortality in Nigeria. Because previous attempts at examining regional disparities in child survival in Nigeria have focused mainly on the period within age 0–59 months, the main focus of the present analysis was the regional disparities in child survival during the two distinct periods of infancy (0–11 months) and childhood (12–59 months).

## Data and Methods

### Data source

This study utilizes the birth recode of the 2008 Nigeria Demographic and Health Survey (NDHS) data. The survey elicited information on demographic and health indicators. The primary sampling unit (PSU), which was regarded as a cluster for the 2008 NDHS, was defined on the basis of enumeration areas (EAs). The sample for the survey was selected using a stratified two-stage cluster design consisting of 888 clusters (NPC & ICF Macro, 2009). In all, a nationally representative sample of 36,800 households was selected for the survey. The community-level variables were measured at the level of the PSU, which serves as a proxy for community or neighbourhood. The PSUs refer to small and administratively defined areas and one PSU is a cluster consisting of at least 80 fairly homogenous households or units. In the 2008 NDHS, data were collected in 886 PSUs, while two PSUs could not be accessed due to disturbances in the areas. A full report of the data collection procedures for the 2008 NDHS is available elsewhere (NPC & ICF Macro, 2009).

In the 2008 NDHS, birth history data were collected from 33,385 women aged 15–49 years. These included sex of child, month and year of child's birth, child's survivorship status, child's current age and age at death if the child had died. Analysis for the present paper was restricted to 28,647 live-born children produced by 18,028 women within the five years before the survey. Analysis was restricted to births within the five years preceding the survey to obtain a picture of the current situations in the various geo-political regions of the country. However, severe omission of births and deaths as well as displacement of dates of those events could seriously affect mortality estimates. Meanwhile, the data quality assessment of the 2008 NDHS indicates that the percentage of missing information regarding births, deaths, birth dates as well as age at death across various characteristics of mothers such as mother's education and region of residence only varied between less than 1% and 3% (NPC & ICF Macro, 2009). This suggests that there was neither a serious under-reporting of infant and child deaths nor serious displacement of information on such vital events that could seriously affect the mortality estimates across regions or across any other mother-level characteristic. In addition, to ensure national representativeness, weighting factors were applied to adjust for oversampling of some locations and under-sampling of others in the Demographic and Health Survey.

### Ethical considerations

This study was based on secondary analysis of an existing dataset with all participant identifiers removed. The survey instruments received ethical approval from the National Ethics Committee in the Federal Ministry of Health, Abuja, Nigeria, and from the Ethics Committee of the Opinion Research Corporation of Macro International Inc., Calverton, MD, USA. Permission to use the 2008 Nigeria DHS data for this study was obtained from ICF Macro Inc.

### Outcome variables

The outcome variables for this study were the risks of death in infancy or childhood, measured as the duration of survival since birth in months. These are defined as either the risk of a child dying between birth and first birthday (infant mortality) or between age 12 and 59 months (child mortality). Analysis was child-based and restricted to the live births in the 5 years before the survey. Hence, all children born within the 5 years before the survey date were included in the analysis. The children's survival status and the age at death in months (if the child had died) or the last month they were known to be alive (if the child was still living at the time of the survey) were combined to generate the outcome variables for the survival analysis. Taking the two durations (0–11 months and 12–59 months) into consideration, children known to have died (i.e. non-censored) were regarded as the cases, whereas children who were still alive at the time of the survey were treated as right-censored (as appropriate for infant and child mortality).

### Exposure variables

#### Community contextual factors

As established in the reviewed literature, the community contextual characteristics of interest in this study included: (1) place of residence, categorized as (i) urban, (ii) rural; (2) region of residence, defined as the regions where the children were raised, and categorized as (i) South-West, (ii) North-Central, (iii) North-East, (iv) North-West, (v) South-East, and (vi) South-South; (3) community poverty level, defined as the average poverty level in the community, and categorized as (i) low, (ii) middle and (iii) high. Other contextual characteristics were (4) community maternal level of education, defined as the proportion of mothers who had at least secondary level of education in the community and categorized as (i) low, (ii) middle, (iii) high; (5) community hospital delivery, defined as proportion of mothers who had hospital delivery in the community, and categorized as (i) low, (ii) middle (iii) high; (6) community prenatal care by skilled provider, defined as the proportion of mothers who attended prenatal care by a skilled health provider in the community and categorized as (i) low, (ii) middle (iii) high; (7) proportion with electric connection in the community, defined as the proportion of mothers from households with electric connection in the community and categorized as (i) low, (ii) middle (iii) high; and (8) proportion with piped water in the community, defined as the proportion of mothers from households that had piped water as source of drinking water in the community and categorized as (i) low, (ii) middle (iii) high.

#### Individual-level characteristics

Based on the reviewed literature, the important individual-level (i.e. child-level and mother-level) characteristics considered in this study were as follows: (1) birth order, defined as birth order of the child and categorized as (i) first births, (ii) 2–4 birth order, (iii) birth order 5+; (2) child's sex, defined as sex of the child and categorized as (i) male, (ii) female; (3) birth interval, defined as interval between two births, categorized as (i) less than 24 months, (ii) 24 months or longer; (4) maternal education, categorized as (i) no education, (ii) primary, (iii) secondary and higher; (5) maternal age, grouped as (i) 15–24 years, (ii) 25–34 years, and (iii) 35 years and older; (6) religious affiliation, categorized as (i) Christianity (ii) Islam (iii) and other; (7) place of delivery, defined as place where child was delivered, and categorized as (i) home, (ii) health facility; and (8) wealth index, categorized as (i) poorest, (ii) poorer, (iii) middle, (iv) richer, (v) richest. The wealth index is the proxy indicator for household socioeconomic status. This was derived from the scores allocated to various household possessions. Wealth index was applied in this analysis as a composite index and an indicator of the socioeconomic status of households. This was because the Demographic and Health Survey generally does not collect information on household income or wealth.

### Statistical analysis

Three levels of analysis – univariate, bivariate and multivariate – were employed in data analysis. At the univariate level, descriptive analysis was done and sample characteristics were presented in percentages to show the distribution of respondents by the selected variables. At the bivariate level, cross-tabulation was done and Pearson's chi-squared test was performed to examine relationship between the outcome variables and the selected independent variables. At the multivariate level of analysis, a two-level multilevel Cox proportional regression analysis was performed to examine the effects of individual- and community-level characteristics on child survival during infancy and childhood, and to determine the extent to which characteristics at the individual and community levels explain regional variations in infant and child mortality in Nigeria.

The multilevel Cox proportional hazards model (survival analysis) was employed for multivariate analysis. This was done for two reasons. First, Cox proportional hazards regression analysis is appropriate for analysis of survival data. It is particularly appropriate for handling censored observations. In social science research, censoring occurs when the value of an observation is not fully known. Cox regression analysis allows for the inclusion of censored data and it models censored time-until-event data as a dependent variable where it can be assumed that the covariates have a multiplying effect on hazard rates. Using Cox proportional hazards regression analysis, both the occurrence of childhood mortality and the time when the child died were combined to generate the outcome variables.

The second reason for using multilevel Cox proportional hazards model was to account for the hierarchical structure of the data. The assumption is that children and their mothers (individuals) are nested within households, while households are in turn nested within communities (Harttgen & Misselhorn, 2006). This suggests that children in households with similar characteristics can have different health outcomes when residing in different communities with different characteristics.

Using the multilevel Cox proportional hazards model, the probability of childhood death was regarded as the hazard. The hazard was modelled using the following equations:
(1)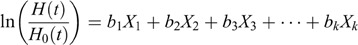, where *X*_1_ … *X_k_* are a collection of explanatory variables and *H*_0_(*t*) is the baseline hazard at time *t*, representing the hazard for a person with the value 0 for all the explanatory variables. By dividing both sides of equation (1) by *H*_0_(*t*) and taking logarithms, equation (1) becomes:
(2)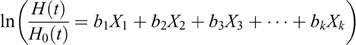, where *H*(*t*)/*H*_0_(*t*) is regarded as the hazard ratio. The coefficients *b_i_* … *b_k_* are estimated by Cox regression.

To estimate both the fixed and random effects in the multilevel survival analysis, it could be assumed that the hazards of any two units are proportional (Rabe-Hesketh *et al.*, 2004) and this can be modelled as: (3)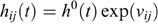.

In equation (3) above, there are two levels (the two subscripts): *i* represents the level 1 units (individuals), *j* stands for the level 2 units (communities) and ν_*ij*_ denotes the linear predictor of the generalized linear latent and mixed model (GLLAMM).

Further, to examine how individual- and community-levels determinants influence survival chances during infancy and childhood, separate models were fitted for infant mortality and child mortality. Fourteen models were fitted in all (seven models each for the two outcome variables). The first model (Model 0 or empty model) contained no explanatory variables, but was fitted to decompose the total variance into its individual- and community-level components. The second model (Model 1) considered only the region of residence covariate in order to examine the independent influence of the region where children were born or raised on their survival chance. The third model (Model 2) incorporated the child-level variables into the multilevel analysis. While the fourth model (Model 3) incorporated the mother-level variables, the fifth model (Model 4) considered only the community-level variables in order to examine the effect of community-level factors on child survival, independent of other factors. The sixth model (Model 5) is the full model that incorporated all the selected variables into the multilevel analysis. The seventh model (Model 6) is the final model. Fitting the final model involved two steps. First, stepwise survival analysis was done to determine the key variables associated with infant and child mortality. Second, all the variables selected from the stepwise Cox regression models were incorporated into the multilevel modelling. All analysis was done using Stata (version 11.2).

GLLAMM – a downloadable program and implementable in Stata – was used to conduct all the multilevel analyses. Fixed effects and random effects, which are important concepts in multilevel analysis, were employed in results interpretation. While fixed effects are used to model associations, random effects are useful in modelling variations (Merlo *et al.*, 2005, 2006). Conventionally, measures such as regression coefficients, odds ratios and hazard ratios are useful measures of association, but these give no information on the health variations within and between populations. Thus in multilevel modelling, measures of variations such as variance partition coefficient (or intra-class correlation) and proportional change in variance are good measures that provide good understanding of contextual determinants of individual health (Merlo *et al*., 2005). In this study, measures of variation represent the extent to which children raised in the same neighbourhood or community are exposed to the same situations such as availability (or non-availability) of health services, medical personnel, electricity, drinkable water and others.

Measures of association (i.e. fixed effects) were expressed in this study as hazard ratios (HRs) and *p*-values. The random effects, which measure variations in infant and child mortality across communities, were expressed in this study as intra-class correlation (ICC) (or variance partition coefficient, VPC), and proportional change in variance (PCV). The intra-class correlation is an important measure of the relatedness of clustered data within community or household units (Antai, 2011b). The VPC was calculated in this study using the linear threshold model method whereby VPC corresponds to the intra-class correlation (Merlo *et al.,* 2005). Hence, the VPC was computed using: 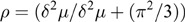, where, ρ is the ICC, δ^2^μ is the variance at the community level, π^2^/3=3.29 and represents the fixed variance at individual level (Merlo *et al.*, 2005). The precision of random effects was determined by the standard error (SE) of the covariates. To determine the goodness-of-fit of the consecutive models, regression diagnostic was done using Akaike Information Criteria (AIC). Boco (2010) noted that a lower value of AIC indicates a better fit.

## Results

### Individual-level characteristics by region of residence

The distribution of the study sample by individual-level characteristics and according to region of residence is presented in Table 1. Huge differences exist in the selected characteristics between regions. With the exception of child's sex, all the selected characteristics vary significantly across the regions of residence (*p*<0.001). With respect to birth order, the proportion of children of birth order five or higher was highest in the North-East (43.2%) and North-West (42.0%) and lowest in the South-West (19.8%) regions. The majority of the children were born to uneducated mothers in the North-Central (44.5%), North-East (73.7%) and North-West (78.4%), while the majority of the children were born to women with secondary or higher education in the three southern regions. Overall, about half of the children were born to uneducated mothers in Nigeria (46.5%). More than nine in ten children were born to Muslim mothers in North-West while over 95% of the children were born to Christian mothers in the South-East and South-South. The proportion of children born to mothers in the poorest households ranged from 4.9% in the South-West to 47.0% in the North-East. The percentage of births delivered in a health facility ranged from 8.6% in the North-West to 77.8% in the South-East.

**Table 1.** Percentage distribution of child- and mother-level characteristics by region of residence, Nigerian DHS 2008

Variable/category	Nigeria % (*n*)	North-Central % (*n*)	North-East % (*n*)	North-West % (*n*)	South-East % (*n*)	South-West % (*n*)	South-South % (*n*)	*p*-value
Region of residence	100	17.6	22.9	27.7	8.6	11.6	11.7	
	(28,647)	(5046)	(6559)	(7947)	(2450)	(3318)	(3327)	
Child's sex								0.950
Male	50.9	51.3	50.3	50.6	51.2	51.0	51.1	
Female	49.1	48.7	49.7	49.4	48.8	49.0	48.9
Birth order								<0.001
First birth	18.7	19.0	15.5	16.2	22.1	24.4	22.1
2–4	45.6	47.6	41.3	41.8	47.9	55.8	48.3
5+	35.7	33.4	43.2	42.0	30.0	19.8	29.5
Birth interval								
<24 months	19.2	15.9	21.2	20.6	26.8	12.3	19.6	
24+ months	80.8	84.1	78.8	79.4	73.2	87.7	80.4	
Maternal education								<0.001
None	46.5	44.5	73.7	78.4	7.2	14.9	6.7
Primary	23.2	29.4	16.6	13.3	31.2	28.9	35.7
Secondary or higher	30.3	26.1	9.6	8.4	61.6	56.2	57.6
Maternal age								<0.001
15–24	24.8	26.1	29.9	30.1	16.5	16.3	20.9
25–34	49.8	49.6	45.8	44.4	55.2	57.0	55.2
35+	25.4	24.4	24.3	25.4	28.3	26.7	23.9
Religion								<0.001
Christianity	43.1	55.3	15.3	4.9	95.7	60.8	95.3
Islam	55.4	42.9	83.6	93.9	0.3	38.4	2.7
Other	1.6	1.8	1.1	1.1	4.0	0.8	1.9
Wealth index								<0.001
Poorest	23.2	23.6	47.0	32.3	5.4	4.9	7.2
Poorer	22.8	24.9	24.5	33.2	10.8	11.3	16.5
Middle	19.3	25.1	16.3	17.8	25.5	13.5	22.8
Richer	17.8	15.0	9.4	10.9	31.4	25.3	28.2	
Richest	16.9	11.4	2.7	5.8	26.9	45.0	25.3	
Place of delivery								<0.001
Home	63.9	58.3	87.1	91.4	22.2	24.3	50.2	
Health facility	36.1	41.7	12.9	8.6	77.8	75.7	49.8	

### Community-level contextual characteristics by region of residence

The distribution of the study sample by community-level characteristics, as shown in Table 2, indicates that all the selected community-level characteristics vary significantly across regions (*p*<0.001). Most of the children were living in rural areas in all the regions, except in the South-West where 55.2% of the children were living in urban areas. Considering how ethnically diverse the six regions are, the results show that the North-Central (69.8%), North-East (62.7%) and South-South (53.7%) were highly heterogeneous; while the South-East (7.2%), North-West (7.8%) and South-West (12.3%) regions were less heterogeneous. Considering education context, the majority of the children in the North-West (70.5%) and North-East (54.2%) were living in communities where the level of women's education was low, while most children in the southern regions were born in communities that had a high proportion of mothers with secondary or higher education. Table 2 also indicates poor health contexts in the north as the majority of children were born to women residing in communities where there was low utilization of prenatal care service and hospital delivery. Infrastructural context was poor in most regions. In all regions, except South-West, more than half of the children were living in communities where there was low access to electricity and drinkable water. Community poverty level appears high in Nigeria. The percentage of children born to mothers residing in communities with a high proportion of poor households ranged from 23.2% in the South-East to 61.4% in the North-West regions.

**Table 2.** Percentage distribution of community-level characteristics by region of residence, Nigerian DHS 2008

Variable/category	Nigeria %	North-Central %	North-East %	North-West %	South-East %	South-West %	South-South %	*p*-value
Place of residence								<0.001
Urban	29.8	24.5	25.6	17.3	45.2	55.2	27.2	
Rural	70.2	75.5	74.4	82.7	54.8	44.8	72.8	
Ethnic diversity								<0.001
Homogenous	31.7	5.3	12.0	48.6	76.4	28.8	14.2	
Mixed	36.4	24.9	25.3	43.6	16.4	58.9	32.1	
Heterogeneous	31.9	69.8	62.7	7.8	7.2	12.3	53.7	
Community maternal level of education								<0.001
Low	38.7	31.4	54.2	70.5	8.9	14.9	2.1	
Middle	27.6	35.7	22.2	14.3	43.5	33.1	39.1	
High	33.8	32.9	23.6	15.2	47.6	52.0	58.9	
Community hospital delivery								<0.001
Low	41.1	27.7	63.6	73.7	5.6	4.8	20.0	
Middle	29.6	31.3	22.4	21.2	36.9	32.6	47.5	
High	29.4	41.0	14.0	5.1	57.5	62.6	32.5	
Community prenatal care by skilled provider								<0.001
Low	43.0	29.2	76.1	68.3	24.6	4.9	16.4	
Middle	26.9	32.0	16.9	21.5	31.2	22.9	48.4	
High	30.1	38.8	7.0	10.2	44.2	72.2	35.2	
Proportion with electric connection in community								<0.001
Low	42.0	59.5	56.8	51.3	19.2	22.0	24.9	
Medium	21.1	19.8	12.6	7.5	46.3	26.4	39.9	
High	36.9	20.7	30.6	41.2	34.5	51.6	35.2	
Proportion with piped water in community								<0.001
Low	63.6	76.9	73.2	61.8	76.7	43.5	57.4	
Medium	4.0	3.5	1.2	2.8	9.0	6.7	3.9	
High	32.4	19.6	25.6	35.4	14.3	49.8	38.7	
Community poverty level								<0.001
Low	22.9	21.1	22.4	12.0	42.7	30.5	28.2	
Middle	33.1	37.3	28.4	26.6	34.1	39.9	40.8	
High	44.0	41.6	49.2	61.4	23.2	29.6	31.0	

### Percentage distribution of infant and child mortality by region of residence

Table 3 presents the bivariate results of the relationship between infant and child mortality and region of residence. The results indicate a significant variation in infant and child mortality across the six regions of the country (*p*<0.001). The table shows that infant mortality was highest in the North-East and South-East (8.0% each), while it was lowest in the South-West (5.2%). Similarly, the results in Table 3 indicate that child mortality was highest in the North-West (6.0%) and lowest in the South-West (1.4%). The distribution of infant and child mortality across the six regions suggests that under-five death was generally more pronounced during infancy than in childhood.

**Table 3.** Percentage distribution of infant and child mortality by region of residence, Nigerian DHS 2008

Region*	Infant survival status		Child survival status	
	Alive (*n*=26,605)	Died (_1_*q*_0_) (*n*=2042)	Alive (*n*=25,446)	Died (_4_*q*_1_) (*n*=1159)
South-West	94.8	5.2	98.6	1.4
North-Central	92.9	7.1	96.8	3.2
North-East	92.0	8.0	95.2	4.8
North-West	92.5	7.5	94.0	6.0
South-East	92.0	8.0	96.9	3.1
South-South	93.1	6.9	97.2	2.8
Nigeria	92.9	7.1	96.0	4.0

* *p*<0.001.

### Risk factors of infant and child mortality: multilevel analysis

**Fig. 1.**
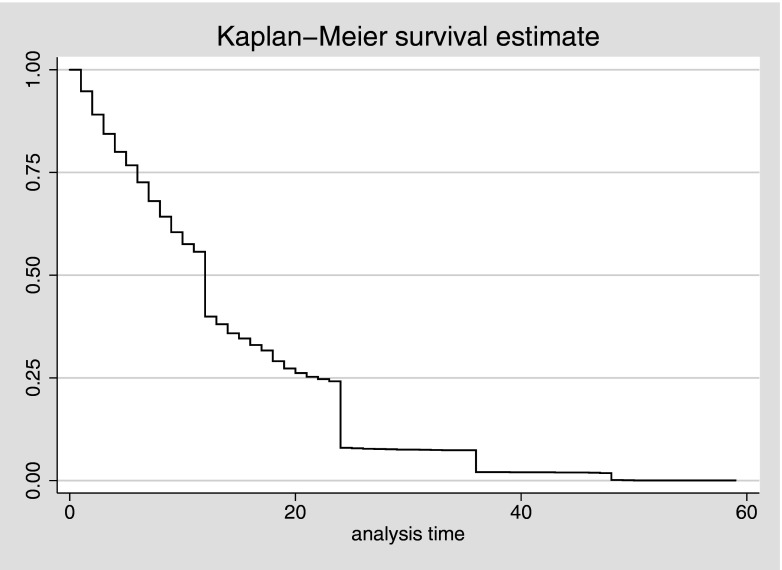
Child survival plot for all children that died before reaching their fifth birthday (duration of survival since birth in months, five years before the survey), Nigeria DHS 2008.

**Fig. 2.**
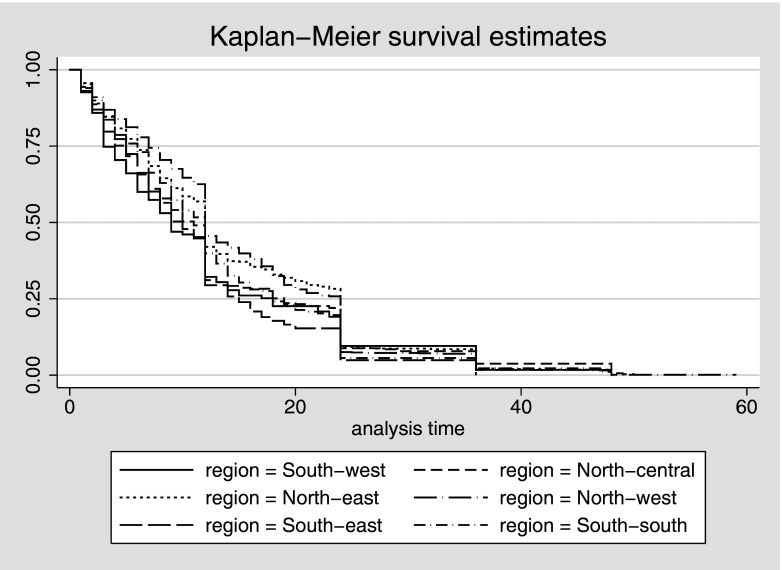
Child survival plots for children that died before reaching their fifth birthday (duration of survival since birth in months, five years before the survey), by region of residence, Nigeria DHS 2008.

The results show that out of a total number of 3201 under-five deaths that occurred during the five years before the survey, 2042 children died during the first year of life while 1159 children died during infancy (Table 3). Figure 1 presents the child survival plot showing the duration of survival since birth for all the children that died before reaching their fifth birthday (0–59 months). Further description of the mortality risks among the children by region of residence is provided by Fig. 2. This figure shows the survival functions of all the children that died during the first five years of life by region.

The results of the null model from the multilevel analysis (Model 0, which contained no explanatory variable) showed a significant variation in infant mortality (Table 4) and child mortality (Table 5) at individual and community levels. The results reveal that the intra-class correlations (ICCs) of 9.6% and 8.4% were associated with infant mortality at the individual and community levels, respectively, as against ICCs of 4.9% and 10.4% for child mortality at individual and community levels, respectively. Model 1 in Tables 4 and 5 shows the effects of the region of residence covariate. Only the region covariate was included in the multilevel model to examine whether risks of infant and child mortality vary across regions. The findings reveal that children in the South-East (HR: 1.65, *p*<0.05), North-East (HR: 1.54, *p*<0.05), North-West (HR: 1.43, *p*<0.05), South-South (HR: 1.41, *p*<0.05) and North-Central (HR: 1.35, *p*<0.05) had significantly higher risks of dying in infancy compared with children in the South-West region. Similarly, the results of Model 1 (Table 5) indicate an elevated risk of death during childhood for children in the North-Central (HR: 1.91, *p*<0.05), North-East (HR: 3.04, *p*<0.05), North-West (HR: 3.74, *p*<0.05), South-East (HR: 1.89, *p*<0.05) and South-South (HR: 1.75, *p*<0.05) compared with children in the South-West region.

**Table 4.** Child- and mother-level compositional and community contextual factors associated with infant mortality in Nigeria, DHS 2008

	Model 0	Model 1	Model 2	Model 3	Model 4	Model 5	Model 6
Characteristic	Empty model (HR)	Region covariate (HR)	Child-level variables (HR)	Mother-level variables (HR)	Community-level variables (HR)	Full model (HR)	Final model (HR)
**Fixed effects**							
Region of residence							
South-West		1	1	1	1	1	1
North-Central		1.35*	1.29*	1.23	1.10	1.22	1.28
North-East		1.54*	1.35*	1.30	1.17	1.27	1.37*
North-West		1.43*	1.22*	1.18	1.12	1.09	1.19
South-East		1.65*	1.49*	1.43*	1.56*	1.29	1.30*
South-South		1.41*	1.33*	1.26*	1.27*	1.32	1.29
Child's sex							
Male			1			1	1
Female			0.79*			0.78*	0.78*
Birth order							
First birth			1			1	1
2–4			1.23*			0.90	0.72*
5+			4.06*			1.20	1.23
Birth interval							
<24 months			1			1	1
24+ months			0.56*			0.50*	0.52*
Maternal education							
None				1		1	1
Primary				0.87		0.88	0.95
Secondary or higher				0.84*		0.84*	0.84*
Maternal age							
15–24				1		1	1
25–34				0.57*		0.77***	0.77*
35+				0.60*		0.85*	0.85*
Religion							
Christianity				1		1	
Islam				0.88		0.78	
Other				0.95		1.03	
Wealth index							
Poorest				1		1	1
Poorer				1.03		0.98	0.97
Middle				0.95		1.06	0.91
Richer				0.89		1.05	0.87
Richest				0.82*		0.80	0.72*
Place of delivery							
Home				1		1	1
Health facility				0.47*		0.55*	0.41*
Place of residence							
Urban					1	1	1
Rural					1.12	1.10	1.22*
Ethnic diversity							
Homogenous					1	1	
Mixed					0.95	0.81*	
Heterogeneous					1.03	0.84	
Community maternal level of education							
Low					1	1	
Medium					0.90	0.96	
High					0.92	0.10*	
Community prenatal care by skilled provider							
Low					1	1	
Medium					0.79*	1.13	
High					0.75*	1.25	
Proportion with electric connection in community							
Low					1	1	
Medium					1.03	0.97	
High					0.94	0.91	
Proportion with piped water in community							
Low					1	1	1
Medium					0.70*	0.79	0.67*
High					0.84*	0.93	0.87*
Community hospital delivery							
Low					1	1	1
Medium					0.90	0.90	0.86
High					0.74*	0.80*	0.70*
Community poverty							
Low					1	1	
Medium					1.08	1.05	
High					1.15	1.06	

**Random effects**	Empty	Region	Child	Mother	Community	Full model	Final model
Community level							
Variance (SE)	0.30 (0.13)*	0.25 (0.09)*	0.22 (0.10)	0.20 (0.08)*	0.17 (0.08)*	0.14 (0.06)*	0.10 (0.06)*
VPC=ICC (%)	8.4	7.1	6.3	5.7	4.9	4.1	2.9
Explained variation (i.e. PCV in %)	Ref.	16.7	26.7	33.3	43.3	53.3	66.7
Individual level							
Variance (SE)	0.35 (0.14)*	0.34 (0.14)*	0.24 (0.11)*	0.23 (0.14)*	0.23 (0.15)	0.15 (0.08)	0.12 (0.07)*
VPC=ICC (%)	9.6	9.4	6.8	6.5	6.5	4.4	3.5
Explained variation (i.e. PCV in %)	Ref.	2.9	28.6	34.3	34.3	57.1	65.7
Log likelihood	−7400.2	−7387.5	−6557.2	−2984.6	−7359.4	−2978.6	−2985.0
Model fit statistics							
AIC	14806.3	14790.9	13146.5	6027.3	14766.8	6047.1	6022.1

HR, hazard ratio; SE, standard error; VPC, variance partition coefficient; ICC, intra-community correlation coefficient; PCV, proportional change in variance; AIC, Akaike information criterion. Ref., reference.

*p<0.05.

**Table 5.** Child and mother-level compositional and community contextual factors associated with child mortality in Nigeria, DHS 2008

	Model 0	Model 1	Model 2	Model 3	Model 4	Model 5	Model 6
Characteristic	Empty model (HR)	Region covariate (HR)	Child-level variables (HR)	Mother-level variables (HR)	Community-level variables (HR)	Full model (HR)	Final model (HR)
**Fixed effects**		
Region of residence							
South-West		1	1	1	1	1	1
North-Central		1.91*	1.86*	1.82	1.62*	2.14*	1.45*
North-East		3.04*	2.80*	2.13*	2.24*	2.38*	1.79*
North-West		3.74*	3.46*	3.32*	2.43*	2.99*	2.00*
South-East		1.89*	1.88*	1.94	1.76*	1.52	1.68*
South-South		1.75*	1.72*	1.26	1.49*	1.26	1.52*
Child's sex							
Male			1			1	1
Female			0.97			0.73*	0.99
Birth order							
First birth			1			1	
2–4			1.53*			1.15	
5+			5.00*			1.31	
Birth interval							
<24 months			1			1	1
24+			0.66*			0.68*	0.59*
Maternal education							
None				1		1	
Primary				1.17		1.11	
Secondary or higher				1.14		1.07	
Maternal age							
15–24				1		1	1
25–34				1.26		1.25	1.10
35+				1.74*		1.71*	1.29*
Religion							
Christianity				1		1	
Islam				0.90		0.80	
Other				0.56		0.63	
Wealth index							
Poorest				1		1	1
Poorer				0.87		0.85	0.88
Middle				1.02		0.97	0.93
Richer				0.82		0.80	0.81
Richest				0.76		0.76	0.62*
Place of delivery							
Home				1		1	1
Health facility				0.68		0.68	0.75*
Place of residence							
Urban					1	1	1
Rural					1.30*	1.33	1.40*
Ethnic diversity							
Homogenous					1	1	1
Mixed					1.05	0.79	1.04
Heterogeneous					0.97	0.59*	0.86
Community maternal level of education							
Low					1	1	
Medium					1.06	1.28	
High					0.97	1.31	
Community prenatal care by skilled provider							
Low					1	1	
Medium					0.96	1.05	
High					0.95	1.12	
Proportion with electric connection in community							
Low					1	1	
Medium					1.06	1.26	
High					0.98	1.16	
Proportion with piped water in community							
Low					1	1	1
Medium					1.30	1.00	1.01
High					0.88	0.85	1.42*
Community hospital delivery							
Low					1	1	
Medium					0.83	0.82	
High					0.64*	0.83	
Community poverty							
Low					1	1	1
Medium					1.16	0.87	1.12
High					1.37*	1.01	1.29*

**Random effects**	Empty	Region	Child	Mother	Community	Full model	Final model
Community-level							
Variance (SE)	0.38	0.31	0.27	0.23	0.19	0.11	0.08
	(0.12)*	(0.10)*	(0.07)*	(0.09)*	(0.08)*	(0.02)	(0.03)*
VPC=ICC (%)	10.4	8.6	7.6	6.5	5.5	3.2	2.4
Explained variation (i.e. PCV in %)	Ref.	18.4	28.9	39.5	50.0	71.1	78.9
Child/mother-level							
Variance (SE)	0.17	0.15	0.15	0.12	0.09	0.09	0.06
	(0.08)*	(0.07)*	(0.08)	(0.07)*	(0.04)*	(0.06)	(0.04)
VPC=ICC (%)	4.9	4.4	4.4	3.5	2.7	2.7	1.8
Explained variation (i.e. PCV in %)	Ref.	11.8	11.8	29.4	47.1	47.1	64.7
Log likelihood	−4856.6	−4786.7	−4362.6	−1477.9	−4758.4	−1469.5	−1476.2
Model fit statistics							
AIC	9719.1	9589.4	8757.1	3013.8	9564.7	3029.0	3142.3

HR, hazard ratio; SE, standard error; VPC, variance partition coefficient; ICC, intra-community correlation coefficient; PCV, proportional change in variance; AIC, Akaike information criterion. Ref., reference.

*p<0.05.

Comparing measures of variation in Model 0 and Model 1 (Tables 4 and 5), the results show that inclusion of the region of residence covariate in the multilevel model yielded significant variance across individual and community levels. For instance, as indicated by the proportional change in variance (PCV) in Model 1 in Table 4, 2.9% and 16.7% of the variance in the risks of infant mortality across individual and community levels, respectively, were explained by region of residence.

Selected characteristics of the children were considered in the multilevel analysis in Model 2. Though a slight reduction was observed, the risks of infant mortality (Table 4) and child mortality (Table 5) remained significantly higher in all the other five regions of the country compared with the South-West (*p*<0.05). Similarly, inclusion of mother-level characteristics in Model 3 resulted in a reduction in the risk of infant mortality and child mortality across regions. The results of Model 3 (Table 4) show that the risk of death during infancy remained significantly higher in the South-East (HR: 1.43, *p*<0.05) and South-South (HR: 1.26, *p*<0.05) relative to the South-West region. Also, living in the North-East (HR: 2.13, *p*<0.05) and North-West (HR: 3.32, *p*<0.05) was associated with an elevated risk of death during childhood (Model 3, Table 5). In comparison with Model 1, after incorporating child-level covariates into Model 2 and mother-level variables into Model 3, the measures of variation remained significant across communities, with ICC associated with risk of death in infancy estimated at 6.5% and 5.7% across individual and community levels, respectively (Model 3, Table 4). As indicated by the PCV in Model 3 (Table 4), 34.3% and 33.3% of the variance in the risks of death during infancy across individual and community levels, respectively, could be explained by characteristics at the mother level.

The selected community-level covariates were considered in the multilevel analysis in Model 4. After incorporating only community-level factors into the multilevel models, the results of Model 4 (Table 4) revealed that the risk of death during infancy remained significantly higher in the South-East (HR: 1.56, *p*<0.05) and South-South regions (HR: 1.27, *p*<0.05); while the risk of death during childhood was significantly higher in all the other five regions, compared with the South-West region. Variance that resulted from the inclusion of only community-level characteristics in the multilevel modelling was significant across communities (Model 4, Table 4). Values of ICC of 6.5% and 4.9% across individual and community levels, respectively, were found to be associated with risk of death before age one, while ICC values of 2.7% and 5.5% across individual and community levels, respectively, were associated with the risk of child mortality. The results show that PCVs of 34.3% and 43.3% across individual and community levels, respectively, in the hazards of death before age one could be explained by community-level characteristics (Model 4, Table 4).

While Model 5 (Tables 4 and 5) contained all the selected individual- and community-level factors, variables that were pre-selected in stepwise Cox proportional hazard models were incorporated into the multilevel model in Model 6 (which is the final model). The results of this model (Table 4) indicate that community-level variables, including region of residence, place of residence, community infrastructure and community hospital delivery, as well as individual-level factors such as child's sex, birth order, birth interval, maternal education, maternal age and wealth index, are important predictors of infant mortality in Nigeria. For instance, the results from Model 6 (Table 4) indicate lower risks of death in infancy for children whose mothers had secondary or higher education (HR: 0.84, *p*<0.05) and for children of mothers residing in communities with a high proportion of hospital delivery (HR: 0.70, *p*<0.05). Similarly, the results from Model 6 (Table 5) indicate that birth interval, maternal age, wealth index and place of delivery (i.e. individual-level factors), and region, place of residence, community infrastructure and community poverty level (i.e. community-level factors) are significant predictors of child mortality in Nigeria.

## Discussions

The objectives of this paper were to examine the effects of individual- and community-level characteristics on infant and child mortality and to determine the extent to which characteristics at these levels influence regional variations in infant and child mortality in Nigeria. As has been found in other similar studies that examined the effects of contextual factors on under-five mortality (Uthman, 2008; Boco, 2010; Antai, 2011b), the findings of the present study indicate that risks of death during infancy and childhood were attributable to both individual- and community-level characteristics, and also to the effects of unobserved factors at those levels.

As indicated by the intra-class correlation (i.e. variance partition coefficient) and the explained variations (i.e. proportional change in variance), the findings consistently showed that individual-level factors were more important in explaining regional variations in infant mortality, while community-level characteristics were more important in explaining regional variations in child mortality in Nigeria. Although community factors tend to influence the association between individual-level factors and the two outcome variables – infant and child mortality – the results show that the community-level effects were larger for risks of death during childhood (age 12–59 months) compared with risks of death during infancy (0–11 months). Whitworth & Stephenson (2002) found that village- or community-level factors were more important in accounting for child mortality than mother- or individual-level characteristics. In addition, after adjusting for the effects of selected child-level, mother-level and community-level variables, the findings established higher child mortality clustering at the community level relative to the individual level. Conversely, higher infant mortality clustering was found at the individual level compared with the community level. This result suggests that community-level attributes appear to play a more important role in child survival during childhood than in infancy. A plausible explanation for this is that children's interaction with community environment or neighbourhood contexts is likely to be higher during ages 12–59 months compared with the period under age one (Boco, 2010).

Further, many of the characteristics at the individual and community levels considered in this study were found to be significantly associated with infant and child mortality. For instance, the region of residence where children were raised tends to affect children survival chances. As previously observed elsewhere by Sastry (1996), community characteristics appear to mitigate infant and child mortality risks in the South-West region of Nigeria, while community characteristics tend to exacerbate infant and child mortality risks in other regions of the country. For example, after incorporating the selected community contextual factors into the multilevel analysis, the results indicated elevated risks of child mortality in the North-Central, North-East, North-West, South-East and South-South regions relative to South-West region.

This result suggests that residence in a particular region of Nigeria is a major determinant of infant and child mortality in the country. This finding may be attributed to spatial inequality in social and economic development between regions (Antai, 2011b), inequality in the distribution and use of health facilities (Stock, 1983; Adetunji, 1994; Pradhan *et al.*, 2003; Say & Raine, 2007), differences in maternal education between and across regions, differences in hygiene practices (Ladusingh & Singh, 2006), differences in age at first marriage (Wall, 1998) as well as differences in community education and use of preventive health care services (Kravdal, 2004; Ladusingh & Singh, 2006). In addition, other causal mechanism such as regional variations in immunization coverage could be responsible for the huge disparities in infant and child mortality between the south and north of Nigeria. For instance, while studies have indicated increase in immunization coverage in Nigeria over the last two decades (Fatiregun & Okoro, 2012; Adegboye *et al.*, 2013), other evidence has suggested a widening gap and variation in the immunization coverage between the south and north of the country, with the former having higher full immunization coverage compared with the latter (Salako & Oluwole, 2009). It has also been established that improvement in women's education, particularly in the northern part of Nigeria, will lead to further reduction in childhood deaths in Nigeria (Adebowale *et al.*, 2012).

Apart from region of residence, other community-level characteristics found to exert significant effects on infant and child mortality in Nigeria include place of residence, access to drinkable water in the community, community hospital delivery and community poverty level. Considering place of residence for instance, infant and child mortality is likely to be higher in the regions that are predominantly rural compared with the more urbanized regions. This is perhaps partly due to the differentials in the distribution of health care facilities between rural and urban communities (Stock, 1983; Adetunji, 1994). Our descriptive findings indicate that more than half of the respondents in the South-West were resident in urban areas, while between 55 and 83% of the people in other regions were residing in rural areas. This huge differential in the composition of rural–urban population across regions seems to contribute to regional variations in infant and child mortality in Nigeria. Also, individual-level attributes such as child's sex, birth order, maternal education, maternal age, wealth index and place of delivery were important predictors of infant and child mortality in Nigeria. For instance, the children of mothers aged 25–34 had lower risks of death compared with children of younger mothers, thus lending credence to the findings of earlier studies (Larreaa & Kawachib, 2005; Antai, 2011a). Children of mothers aged 25–34 are likely to be better catered for than the children of younger mothers because younger mothers are generally inexperienced about child rearing, they are mostly uneducated and often marry too early. Wall (1998) cited the challenges facing young mothers as child's death, severe anaemia, pregnancy-induced hypertension, obstructed labour, haemorrhage, fistula and even death.

The findings of this paper suggest that regional variations in the risks of death during infancy and childhood were largely attributable to characteristics at the individual level as well as the community characteristics affecting the context in which children are raised. Thus, this study has established that individual- and community-level factors are important in explaining regional variations in infant and child mortality in Nigeria.

These findings have important policy implications. In order to address regional disparities in infant and child mortality in Nigeria, it is important to look beyond individual-level attributes. Contextual characteristics of the community or neighbourhood must also be addressed. Social, economic and health outcome disparities have been largely attributed to differences in regional distributions of social services, health facilities, housing conditions and other essential services (Adetunji, 1994; Jatrana, 2003; Larreaa & Kawachib, 2005). Hence, efforts must be intensified by both central and regional or state governments to address the imbalances in socioeconomic development across the six regions of Nigeria, as well as the intra-region spatial inequality in social and economic development across communities within the various regions of the country. Further, the Kaplan–Meier survival curves (Fig. 2) and descriptive findings in this study suggest that regional disparities in under-five mortality are more pronounced during infancy as well as during the period before age two. Policies that address regional disparities in under-five mortality in Nigeria must include strategies to improve child health outcomes, particularly during the first year of life.

This study has its limitations. First, it adopted primary sampling units (PSUs) as a proxy for community or neighbourhood. This may generate information biases due to misclassification of respondents into wrong administrative boundaries (Antai, 2011a). Second, with the exception of the region of residence and place of residence covariates, other community-level variables in this study were generated by aggregating the individual-level variables at the level of PSUs. This process could lead to auto-correlation of generated community-level variables with any of the individual- or household-level characteristics. Meanwhile, to minimize this problem, a correlation test of all the selected variables was conducted, and no two variables were found to be highly correlated. Nevertheless, to provide better estimates of influence of community-level characteristics on child survival, it is recommended that future studies consider using census data and other surveys that collect distinct community-level variables. Third, other important contextual factors (such as proximity to health facility, cultural practices and customs) could not be addressed in this study. Being an analysis of a secondary dataset, such important factors were unavailable in the DHS dataset. As a result, further studies on the contextual factors associated with infant and child mortality are needed in Nigeria. The temporal sequence of events for some variables may also pose some limitation. Nonetheless, the study has its own strengths. First, it provides empirical evidence that better understanding of the characteristics at both individual- and community-levels is crucial for addressing regional variations in infant and child mortality in Nigeria. Second, DHS datasets are nationally representative and the findings could easily be generalized across the whole country. Also, international comparisons of the results are possible, because DHS surveys adopt similar instruments across countries.

In conclusion, this study has demonstrated the importance of both individual and community or neighbourhood contexts in explaining regional variations in infant and child mortality in Nigeria. The results of this study underscore the need to look beyond the influence of individual-level factors in addressing the regional variations in infant and child mortality in the country. In order to ensure substantial reduction in under-five mortality during infancy and childhood, attention needs to be focused on community-level interventions aimed at improving child survival in the country's socially and economically disadvantaged areas.

## References

[ref1] Adebowale A., Yusuf B. & Fagbamigbe A. (2012) Survival probability and predictors for woman experience childhood death in Nigeria: analysis of north–south differentials. BMC Public Health 12(1), 430. 22691616 10.1186/1471-2458-12-430 PMC3432604

[ref2] Adegboye O. A., Kotze D. & Adegboye O. A. (2013) Multi-year trend analysis of childhood immunization uptake and coverage in Nigeria. Journal of Biosocial Science doi:10.1017/S0021932013000254 10.1017/S0021932013000254 23710666

[ref3] Adekanmbi V. T., Kayode G. A. & Uthman O. A. (2013) Individual and contextual factors associated with childhood stunting in Nigeria: a multilevel analysis. Maternal and Child Nutrition 9(2), 244–259. 22004134 10.1111/j.1740-8709.2011.00361.x PMC6860873

[ref4] Adetunji J. A. (1994) Infant mortality in Nigeria: effects of place of birth, mother's education and region of residence. Journal of Biosocial Science 26(4), 469–477. 7983098 10.1017/s002193200002160x

[ref5] Adetunji J. A. (1995) Infant mortality and mother's education in Ondo State, Nigeria. Social Science & Medicine 40(2), 253–263. 7899937 10.1016/0277-9536(94)e0067-3

[ref6] Antai D. (2009) Inequitable childhood immunization uptake in Nigeria: a multilevel analysis of individual and contextual determinants. BMC Infectious Diseases 9, 181. 19930573 10.1186/1471-2334-9-181 PMC2787508

[ref7] Antai D. (2011a) Inequalities in under-5 mortality in Nigeria: do ethnicity and socioeconomic position matter? Journal of Epidemiology 21(1), 13–20. 20877142 10.2188/jea.JE20100049 PMC3899512

[ref8] Antai D. (2011b) Regional inequalities in under-5 mortality in Nigeria: a population-based analysis of individual- and community-level determinants. Population Health Metrics 9(6), 1–27. 21388522 10.1186/1478-7954-9-6 PMC3065413

[ref9] Antai D., Wedrén S., Bellocco R. & Moradi T. (2009) Ethnic disparities in child health in Nigeria: a multilevel analysis of individual and contextual factors. Ethnicity and Inequalities in Health and Social Care 2(4), 39–49.

[ref10] Aremu O., Lawoko S., Moradi T. & Dalal K. (2011) Socio-economic determinants in selecting childhood diarrhoea treatment options in Sub-Saharan Africa: a multilevel model. Italian Journal of Paediatrics 37(13), 2–8. 10.1186/1824-7288-37-13 PMC3071781 21429217

[ref11] Babalola S. & Fatusi A. (2009) Determinants of use of maternal health services in Nigeria – looking beyond individual and household factors. BMC Pregnancy and Childbirth 9(1), 43. 19754941 10.1186/1471-2393-9-43 PMC2754433

[ref12] Black R. E., Morris S. S. & Bryce J. (2003) Where and why are 10 million children dying every year? The Lancet 361, 2226–2234. 10.1016/S0140-6736(03)13779-8 12842379

[ref13] Boco A. G. (2010) Individual and community level effects on child mortality: an analysis of 28 Demographic and Health Surveys in sub-Saharan Africa. DHS Working Papers No. 73 ICF Macro, Calverton, MD, USA.

[ref14] Buor D. (2002) Mothers' education and childhood mortality in Ghana. Health Policy 64, 297–309. 12745169 10.1016/s0168-8510(02)00178-1

[ref15] Diez-Roux A. V., Merkin S. S., Arnett D., Chambless L., Massing M. Nieto F. J. & Watson R. L. (2001) Neighbourhood of residence and incidence of coronary heart disease. New England Journal of Medicine 345(2), 99–106. 11450679 10.1056/NEJM200107123450205

[ref16] Fatiregun A. A. & Okoro A. O. (2012) Maternal determinants of complete child immunization among children aged 12–23 months in a southern district of Nigeria. Vaccine 30(4), 730–736. 22137878 10.1016/j.vaccine.2011.11.082

[ref17] Fayeun O. & Omololu O. (2011) Ethnicity and child survival in Nigeria. African Population Studies 25 (Supplement 1), 92–112.

[ref18] Galster G. C. (2010) The mechanism(s) of neighborhood effects theory: evidence, and policy implications. Paper presented at the ESRC Seminar: Neighbourhood Effects: Theory & Evidence, St Andrews University, Scotland, UK, 4–5th February 2010.

[ref19] Grais R. F., Dubray C., Gerstl S., Guthmann J. P., Djibo A., Nargaye K. D. & Guerin P. J. (2007) Unacceptably high mortality related to measles epidemics in Niger, Nigeria, and Chad. PLoS Med 4(1). 10.1371/journal.pmed.0040016 PMC1761051 17199407

[ref20] Griffiths P., Madise N., Whitworth A. & Matthews Z. (2004) A tale of two continents: a multilevel comparison of the determinants of child nutritional status from selected African and Indian regions. Health & Place 10, 183–199. 15019912 10.1016/j.healthplace.2003.07.001

[ref21] Harttgen K. & Misselhorn M. (2006) A multilevel approach to explain child mortality and undernutrition in South Asia and sub-Saharan. Ibero-America Institute for Economic Research 156, 1–38.

[ref22] Jatrana S. (2003) Infant Mortality in a Backward Region of North India: Does Ethnicity Matter? Asian Metacentre Research Papers.

[ref23] Kravdal Ø. (2004) Child mortality in India: the community-level effect of education. Population Studies 58(2), 177–192. 15204252 10.1080/0032472042000213721

[ref24] Ladusingh L. & Singh C. H. (2006) Place, community education, gender and child mortality in North-east India. Population, Space and Place 12(1), 65–76.

[ref25] Larreaa C. & Kawachib I. (2005) Does economic inequality affect child malnutrition? The case of Ecuador. Social Science & Medicine 60, 165–178. 15482876 10.1016/j.socscimed.2004.04.024

[ref26] Lawoyin T. O. (2001) Risk factors for infant mortality in a rural community in Nigeria. Journal of Royal Society for the Promotion of Health 121(2), 114–118. 10.1177/146642400112100213 11467203

[ref27] Macintyre S., Ellaway A. & Cummins S. (2002) Place effects on health: how can we conceptualise, operationalise and measure them? Social Science & Medicine 55, 125–139. 12137182 10.1016/s0277-9536(01)00214-3

[ref28] Manda S. O. M. (2001) A comparison of methods for analysing a nested frailty model to child survival in Malawi. Australian & New Zealand Journal of Statistics 43(1), 7–16.

[ref29] Merlo J., Chaix B., Ohlsson H., Beckman A., Johnell K., Hjerpe P. & Larsen K. (2006) A brief conceptual tutorial of multilevel analysis in social epidemiology: using measures of clustering in multilevel logistic regression to investigate contextual phenomena. Journal of Epidemiology and Community Health 60, 290–297. doi: 10.1136/jech.2004.029454 16537344 PMC2566165

[ref30] Merlo J., Chaix B., Yang M., Lynch J. & Rastam L. (2005) A brief conceptual tutorial of multilevel analysis in social epidemiology: linking the statistical concept of clustering to the idea of contextual phenomenon. Journal of Epidemiology and Community Health 59, 443–449. doi: 10.1136/jech.2004.023473 15911637 PMC1757045

[ref31] NPC & ICF Macro (2009) Nigeria 2008 Demographic and Health Survey. National Population Commission (NPC) [Nigeria] & ICF Macro, Calverton, MD, USA.

[ref32] NPC & ORC Macro (2004) Nigeria 2003 Demographic and Health Survey. National Population Commission (NPC) [Nigeria] & ORC Macro, Calverton, MD, USA

[ref33] Odimegwu C. O. (2002) Determinants of breast-feeding status in eastern Nigeria. African Population Studies 17(1), 69–82.

[ref34] Omariba D., Beaujot R. & Rajulton F. (2007) Determinants of infant and child mortality in Kenya: an analysis controlling for frailty effects. Population Research and Policy Review 26, 299–321.

[ref35] Pradhan M., Sahn D. E. & Younger S. D. (2003) Decomposing world health inequality. Journal of Health Economics 22(2), 271–293. 12606146 10.1016/S0167-6296(02)00123-6

[ref36] Rabe-Hesketh S., Skrondal A. & Picklesz A. (2004) GLLAMM Manual. Division of Biostatistics Working Paper Series, University of California, Berkeley.

[ref37] Rutherford M. E., Mulholland K. & Hill P. C. (2010) How access to health care relates to under-five mortality in sub-Saharan Africa: systematic review. Tropical Medicine & International Health 15(5), 508–519. 20345556 10.1111/j.1365-3156.2010.02497.x

[ref38] Salako A. A. & Oluwole F A. (2009) An appraisal of immunisation in Nigeria: towards improving coverage. Nigerian Hospital Practice 3(3–4).

[ref39] Sastry N. (1996) Community characteristics, individual and household attributes, and child survival in Brazil. Demography 33(2), 211–229. 8827166

[ref40] Sastry N. (1997a) Family-level clustering of childhood mortality risk in northeast Brazil. Population Studies 51, 245–261.

[ref41] Sastry N. (1997b) What explains rural–urban differentials in child mortality in Brazil? Social Science & Medicine 44(7), 989–1002. 9089920 10.1016/s0277-9536(96)00224-9

[ref42] Say L. & Raine R. (2007) A systematic review of inequalities in the use of maternal health care in developing countries: examining the scale of the problem and the importance of context. Bulletin of the World Health Organization 85, 812–819. 18038064 10.2471/BLT.06.035659 PMC2636485

[ref43] Stock R. (1983) Distance and the utilization of health facilities in rural Nigeria. Social Science & Medicine 17(9), 563–570. 8 6879255 10.1016/0277-9536(83)90298-8

[ref44] UNICEF (2012) UNICEF Report on Child Deaths. UNICEF, Geneva.

[ref45] Uthman O. A. (2008) Environmental factors, neighbourhood deprivation, and under-five mortality in Nigeria: an exploratory spatial data analysis. Internet Journal of Pediatrics and Neonatology 9(1), 1–10.

[ref46] Wall L. L. (1998) Dead mothers and injured wives: the social context of maternal morbidity and mortality among the Hausa of northern Nigeria. Studies in Family Planning 29(4), 341–359. 9919629

[ref47] Whitworth A. & Stephenson R. (2002) Birth spacing, sibling rivalry and child mortality in India. Social Science & Medicine 55, 2107–2119. 12409124 10.1016/s0277-9536(02)00002-3

[ref48] WHO (2003) An analytical framework for the study of child survival in developing countries by Mosley W.H and Chen L.C. Bulletin of the World Health Organization 81(2), 140–145. 12756980 PMC2572391

[ref49] Zaba B. & David P. H. (1996) Fertility and the distribution of child mortality risk among women: an illustrative analysis. Population Studies 50(2), 263–278.

